# Physicians’ perceptions of autonomy support during transition to value-based reimbursement: A multi-center psychometric evaluation of six-item and three-item measures

**DOI:** 10.1371/journal.pone.0230907

**Published:** 2020-04-01

**Authors:** Anthony C. Waddimba, David C. Mohr, Howard B. Beckman, Mark M. Meterko

**Affiliations:** 1 Baylor Scott and White Research Institute, Dallas, Texas, United States of America; 2 Department of Surgery, Health Systems Science, Baylor University Medical Center, Dallas, Texas, United States of America; 3 Center for Healthcare Organization and Implementation Research (CHOIR), VA Boston Healthcare System, Boston, Massachusetts, United States of America; 4 Department of Health Law, Policy and Management, Boston University School of Public Health, Boston, Massachusetts, United States of America; 5 Departments of Family Medicine, Internal Medicine & Public Health Sciences, University of Rochester School of Medicine and Dentistry, Rochester, New York, United States of America; 6 Common Ground Health, Rochester, New York, United States of America; 7 Department of Performance Measurement, VA Office of Analytics and Business Intelligence (OABI), Washington, D.C., United States of America; Chinese Academy of Medical Sciences and Peking Union Medical College, CHINA

## Abstract

**Background:**

Successive health system reforms have steadily eroded physician autonomy. Escalating accountability demands placed on physicians concurrent with diminishing autonomy plus widespread “cost cutting” endanger clinical work-life quality and, in turn, threaten patient-care quality, safety, and continuity. This has engendered a renewed emphasis on bettering physician work-life to safeguard patient care. Research indicates that autonomy support could be an effective intervention point in this dynamic, and that improving healthcare practitioners’ experience of autonomy can promote better patient outcomes. New measures of autonomy support towards physicians during systemic/organizational transformation are thus needed.

**Objective:**

We investigated the validity and reliability of two versions of a brief measure of physicians’ perceptions of autonomy support.

**Design:**

Psychometric evaluation of practitioners’ responses to a theory-based, pilot-tested, multi-center, cross-sectional survey-questionnaire.

**Participants:**

Physicians serving in California, Massachusetts, or upstate New York clinical practices implementing pay-for-performance incentives were eligible. We obtained responses from 1,534 (35.14%) of 4,365 physicians surveyed.

**Analysis:**

We randomly partitioned the study sample equitably into derivation and validation subsamples. We conducted parallel analysis, inter-item/point-biserial correlations, and item-response-theory-based graded response modeling on six autonomy support items. Three items with the highest (a) point-biserial correlations, (b) item-level discrimination and (c) information capture were used to construct a short-form (3-item) version of the full (6-item) autonomy scale. We utilized exploratory structural equation modeling and confirmatory factor analysis to establish the factor structure and construct validity of the full-length and short-form scales before comparing their factor invariance, reliability and interrater agreement across physician subgroups.

**Findings:**

All six autonomy support items loaded highly onto one factor accounting for the majority of variance and demonstrating good data fit. The three most discriminating and informative items loaded equally well onto a single factor with similar goodness-of-fit to the data. The three-item scale correlated highly with its six-item parent, showing equally high sensitivity and specificity in discriminating high autonomy support. Variability in scores nested predominantly at within- rather than between-subgroup levels.

**Conclusions and implications:**

Our data supported the factor structure, construct validity, internal consistency, and reliability of six- and three-item autonomy support scales. These brief tools are easily incorporated into multi-dimensional questionnaires at relatively low cost.

## Introduction

Physicians typically are entrusted with a significant level of autonomy,[1, 2] predicated on trust [3] in each individual professional’s competence.[4] The implicit social contract is that, in exchange for self-regulation, physicians will exercise their expertise in a fiduciary manner, respecting each patient’s autonomy,[2, 5] and placing patients’ interests above their proprietary interests.[5–7] In addition, federal/state regulators limit physician autonomy over medical decisions imbued with a high potential for societal harm (e.g. prescribing narcotics).[8] Professional societies exert further constraints by recommending limits to physician ordering practices, as in the *Choosing Wisely* campaign, to reduce “low value” services.[9]

The last thirty years have witnessed a more precipitous erosion of physician autonomy [10, 11] from its peak in the “golden age of doctoring”[12] to new lows in the contemporary “accountable care” era.[13, 14] Escalating constraints on autonomy, instituted in successive waves of health reform (i.e. managed care, evidence-based medicine, patient-centered care, and value-based care) [7, 8, 15–17] are intended to curb unwarranted variations in the quality and cost of medical services.[18] Diminution of autonomy has coincided with greater time pressure at work,[19] cost-saving measures,[20] more stringent licensure/recertification requirements,[21] explosion of clerical tasks,[22] rapid spread of electronic medical records,[23] health information technology systems,[24] and increasing standardization of care as a strategy to improve outcomes.[25] This plethora of changes, often mandated by federal or state regulators, by cumulatively degrading autonomy further, discourage practitioner involvement in quality improvement (QI) projects,[26] and challenge their occupational well-being,[24, 27–29] thereby putting patient-care quality and safety at risk.[30, 31] Attention is thus increasingly shifting to strategies that foster physician well-being [32, 33] (e.g. via enhanced support towards professional autonomy) [34–36] as a core element in improving care quality and safety.[37]

## Theoretical foundations and policy considerations

Professional autonomy is conceptualized in multiple theoretical models as fundamental to engagement and fulfillment at work. Self-determination theory (SDT) considers autonomy as one of three universal, innate psychological needs.[38] In the “areas of work-life” model,[39] job control/autonomy is considered one of six core dimensions of work-life.[40] The job demands/resources [41] and conservation of resources [42] models view autonomy as a resource that can empower resilience among professionals. In a similar fashion, the effort-rewards imbalance model posits that autonomy can shift the balance in favor of job rewards.[43] There is a growing realization that individuals perform optimally in workplaces that are more supportive of their autonomy.[44] Multiple studies, across diverse work domains, confirm that greater autonomy support is positively linked to higher work performance and well-being.[45–48] Motivational strategies, such as pay-for-performance (P4P), that use extrinsic incentives to externally control work behavior are thus questioned by advocates of more autonomous forms of motivation.[49, 50] Proponents of these approaches contend that work organizations achieve better results by “empowering” instead of “overpowering” professionals, and by supporting rather than suppressing professional autonomy.[49, 50] Previous studies from diverse domains have shown that organizations can adapt and their leaders can be trained to provide greater autonomy support.[47, 51] A healthcare organization could, for instance, convene a team of practitioners tasked with finding strategies for reducing post-operative narcotic use. While the task is selected by the organization, an autonomy-supportive approach would let the team brainstorm and determine how best to achieve the desired results. An autonomy-reducing approach would be for organization leaders to craft their own strategy and then require practitioners to operationalize an action plan in whose formulation they did not participate.

In healthcare, practice autonomy is a key ingredient of physicians’ job satisfaction.[28, 52, 53] Experiencing autonomy-supportive mentors during a clinical rotation in medical school makes trainee physicians more likely to choose that specialty.[54] Likewise, young physicians who begin careers in settings with high autonomy support are more inclined to remain practicing there over the long term.[55] High autonomy support is positively associated with greater work-life well-being/satisfaction and negatively associated with intention to leave, suicidal ideation, and job distress.[56] Providers receiving greater support towards their practice autonomy are in turn more supportive of patients’ autonomy.[57] Low autonomy, by contrast, is linked to greater workplace challenges in patient care delivery, and personal challenges such as achieving work-home balance.[43] Additionally, physicians often perceive changes in the financial incentives of reimbursement organizations as a threat to their autonomy.[24, 27]

Collectively these considerations argue for the importance of surveillance measures that reliably track physicians’ experience of autonomy support in their clinical practice. A valid measure of autonomy support could provide a starting point for healthcare organizations aiming to inspire physician buy-in towards QI programs, reduce burnout, and strengthen resilience; thereby improving the clinical workplace climate. Such a tool could also facilitate research into physician autonomy support by, for example, assessing the effect of various organizational transformations on the healthcare workforce.

### The present study

The main goal of this study was to develop and assess the reliability and validity of a measure of physicians’ perceptions of support by the payer organization towards their clinical work autonomy. Lin proposes that physicians exert autonomy over administrative/logistic and/or clinical/knowledge decisions.[58] Salvatore *et al*.’s three-level perspective encompasses (a) clinical work autonomy, (b) social or economic work autonomy, and (c) influence on organizational decisions.[59] In the present study, we focused on support for clinical rather than administrative or social/economic work autonomy. We anchored our framing of autonomy support on these behaviors:[38] acknowledgment of physicians’ perspectives/feelings, providing rationale for any proposed changes, constructive informational feedback to physicians, and overall support towards physicians’ initiatives, choices, and medical decision-making.

Our secondary aim was to empirically extract a shortened version of that measure and to compare the psychometric properties of the short-form scale with that of the full-length version. Thirdly, we aimed to assess potential generalizability of both measures to diverse settings by examining measurement invariance among disparate subgroups of physicians. Fourthly, we aimed to empirically test whether the measures significantly capture collective perceptions [60] of autonomy support at the group/team level or whether they assess the construct only at the individual level.

## Methods

### Study design

This measurement development and psychometric assessment study was based on a secondary analysis of cross-sectional data from surveys of primary care practitioners affiliated with three sites participating in the *Rewarding Results* demonstration project.[61] The Institutional Review Board at Boston University Medical Center approved this multicenter project under Protocol H-26824.

### Selection of study sites

The study utilizes data from three healthcare markets (California, Massachusetts, and upstate New York) that were transitioning to value-based reimbursement for medical services. We selected study sites from a sampling frame of physician organizations in each state that contracted directly with health plans to implement pay-for-performance (P4P) incentives and whose senior executives consented to their organizations’ participation in the survey. Due to the large number of such entities in California and Massachusetts, we divided those lists into diverse strata based on practice size (e.g., <20, 20–69, ≥ 70 physicians), geographic location, insurance type (Medicaid, health maintenance organization, or preferred provider organization) and nature of organization (independent practice association or medical group).[62] We selected entities, for inclusion in the study, randomly from each stratum ensuring that the proportions of primary care physician attributes in each state sample roughly represented their distribution statewide. From California, we sampled a collection of 27 physician groups representative of primary care practices of different sizes, from small to very large, from diverse geographic areas of the state.[62] From Massachusetts, we sampled 26 physician groups from diverse geographic areas of the state whose performance was being profiled by Massachusetts Health Quality Partnership (MHQP).[62] The single New York entity sampled was a partnership between Rochester Independent Physician Practice Association (RIPA) and Excellus-Blue Cross Blue Shield, a Rochester-based health plan that enrolled >70% of the non-Medicare/non-Medicaid (commercial) population across nine counties.[63]

### Study population

In the selected centers, we targeted physicians in three primary care specialties—family practice, general internal medicine, and general pediatrics—who were involved in the local P4P contract for prospective enrollment in the study. Of the eligible physicians, we successfully contacted 1,531 from California; 1,421 from Massachusetts; and 574 from Rochester, NY. Completed questionnaires were received from 1,534: with (response rates) 689 (45%) from California, 554 (39%) from MHQP, and 291 (51%) from RIPA.

### Survey instrument

The multi-dimensional questionnaire used in this study was designed to gather information on physicians’ attitudes towards P4P programs in general, experience-based attitudes toward specific P4P-incentivized clinical guidelines applicable to their patient panel, perceived autonomy support from the health plan(s), and satisfaction with practice. Detailed contents of the survey-questionnaire are described in previous publications.[62–68] A panel of subject matter experts drafted an initial list of candidate items.[64] We used Microsoft Word^®^ software (© Microsoft Corporation, Redmond, WA) to evaluate readability of drafted items based on Flesch-Kincaid grade level and Flesch Reading Ease scores [69]. We aimed for a 7^th^ to 8^th^ grade reading level. To establish *face validity* and *content validity*, candidate items were pilot tested at three Boston-area medical groups (a rural practice of 69, an urban practice of 17, and a suburban practice of 19 physicians). Feedback both from written suggestions and “think aloud” group debriefing sessions was then used to revise and shorten the questionnaire. The final version of the instrument took an average of 15 minutes to complete. The present study focused on autonomy support items in the survey-questionnaire.

### Data collection

Administration of the survey was preceded by various promotional activities intended to maximize participation by the targeted physicians. The study team mailed hard copies of the self-administered survey-questionnaire, together with cover letters, to a physician champion/liaison at each participating organization. The liaison distributed the questionnaires and cover letters to potential respondents either face-to-face at pre-scheduled physician group meetings or by mail via official intra-organization mail systems. Each survey packet also included a prepaid business reply envelope for the return of the completed questionnaire. We continued promoting the survey via monthly telephone calls and mailed reminders to non-respondents. We administered surveys at California and Massachusetts sites from May to November 2004 and, at the Rochester, New York, site from late 2002 through 2003.

### Measures

#### Reference scale

The final questionnaire instrument contained six items addressing physician perceptions of autonomy support (PPAS) from the contracting health plan.[67, 68] We conceptualized physician autonomy as the freedom to use the best professional judgment when applying scientific knowledge and clinical expertise for the good of patients.[8] In the SDT framework, autonomous behavior is that which one engages in of their own volition. From this perspective, autonomy is not seen as synonymous with independence (as in the classic job characteristics/redesign model,[70] for instance) but rather is conceptualized as volitional behavior exercised while staying accountable [71] and within structural boundaries.[72] Items capturing ‘autonomy support’ were derived through an iterative process that began with an extensive literature search focused on studies examining autonomy support among professional employees in diverse work domains. We combined candidate items adapted from the Work Climate Questionnaire [73] and Job Diagnostic Survey [70] with new items crafted by the research team, all reworded to fit the study context. Through the expert panel review and pilot testing described above we reduced the number of autonomy support items to six. The six questions are: Q46 –the health plan *seeks to maintain good relationships with practitioners*, Q47 –the health plan *wants me to take good care of my patients*, Q48 –the health plan *interferes with how I care for my patients*, Q49 –the health plan *understands my situation and needs as a practitioner*, Q50 –the health plan *has confidence in my ability to offer high quality care*, and Q51 –the health plan *encourages my questions and feedback*. Respondents rated the frequency of the experience described using a five-point Likert-style response format as follows: 1 = “None of the time”; 2 = “Rarely”; 3 = “Some of the time”; 4 = “Most of the time”; or 5 = “All of the time”. The scale was scored by first reverse coding the negatively worded item (Q48) before summating then averaging each respondent’s ratings across all six items. Higher scale scores thus indicated higher perceived autonomy support from the health plan.

#### Index scale

Physicians are overwhelmed with requests to complete questionnaires for survey studies.[74] To reduce respondent burden, three-item physician self-report scales have been validated for constructs such as dissatisfaction with practice,[75] discomfort with diagnostic uncertainty,[76] job control,[64] job autonomy,[77] learning opportunities,[77] plus each of three dimensions of burnout.[78] We likewise sought to derive an empirically-supported short-form scale comprised of three of the six PPAS items and to thereafter compare the validity and reliability of this shorter measure, as the index scale, with the fullest measure extractable from all the six items, which we treated as the reference scale.

#### Convergent validity

We explored the subtype of construct validity known as *convergent validity*, i.e. the degree to which measures of constructs that theoretically should be related are, in fact, related. Accordingly, we correlated the six-item and three-item physician perceptions of autonomy support (PPAS-6 and PPAS-3) scales with scales measuring conceptually related constructs such as individual job control, perceptions of peer/staff cooperation, fairness/equity in distribution of P4P bonuses, and global satisfaction with practice. Job control was assessed on a three-item scale (e.g. “Actions necessary to obtain the financial incentive are within my control”).[64, 68] Peer/staff cooperation was assessed on scale comprising two items (e.g. “I am able to obtain the cooperation of other physicians as needed in order to obtain this financial incentive”).[64] We assessed fairness/equity by a single item (“The financial incentive is applied fairly to physicians based on their performance”).[67] Respondents rated their agreement with each item on job control, peer/staff cooperation, and fairness/equity measures on a five-level Likert-type scale ranging from 1 = ‘strongly disagree’ to 5 = ‘strongly agree’. Scores were obtained by averaging the respondent’s ratings. Global job satisfaction was assessed using a single item: “Overall, how satisfied are you with your current medical practice?”, and participants responded by selecting a rating on a seven-point Likert-style scale ranging from 1 = “completely dissatisfied” to 7 = “completely satisfied.”

#### Discriminant validity

We likewise sought to demonstrate the subtype of construct validity referred to as *discriminant validity*, i.e. the degree to which measures of constructs that theoretically are not supposed to be related are, in fact, unrelated. We thus tested correlations of both the six-item and three-item perceived autonomy support scales with two measures of perceptions deemed to be antithetical to experiences of autonomy support from their respective payor organizations: (1) a single-item measure of the perceived difficulty of the incentivized clinical task, and (2) physician perceptions of whether P4P incentives hindered patient care. The clinical task difficulty item was phrased as follows: “It is more difficult for me to obtain this financial incentive than it is for other physicians”.[68] Perceived hindrance of patient care was likewise assessed by a single item: “Physician efforts to achieve quality targets hinder them providing other essential medical services”.[68] Respondents rated their agreement with each item along a 5-level Likert scale format ranging from 1 = ‘strongly disagree’ to 5 = ‘strongly agree’.

### Analytic strategy

We randomly split the overall study dataset equitably into a derivation sample (n = 767) and a validation sample (n = 767). We assessed distribution of physician, practice, and study site characteristics in both subsets to confirm that random partitioning was successful.

We derived Bartlett’s test of sphericity [79] and the Kaiser-Meyer-Olkin (KMO) test [80] to obtain empirical support for adequacy of the study data as a source of factor-analytic correlation matrices. The null hypothesis for Bartlett's test of sphericity is that the correlation matrix of the study variables does not significantly differ from an identity matrix, which would imply that the variables are, in fact, unrelated and thus unsuitable for structure detection.[79] The KMO measure of sampling adequacy assesses the proportion of variance in study variables that might be due to underlying factors.[80] High values (close to 1.0) suggest that a factor analysis of study data will yield meaningful findings, while values less than 0.50 indicate that results of a factor analysis might not be useful. One rule of thumb is that a KMO ≥ 0.80 strongly indicates that there are sufficient indicator items for each hypothesized factor to be extracted.[80] Eigenvalues relate to the proportion of the variance in the data that the extracted factors explain or account for. One rule of thumb is that extracted factors should collectively account for more than 60% of the total variance.[81]

We utilized the method of Mundfrom *et al* [82] to compute minimum sample size requirements for factor analyses. Number of variables to factors (*p/f*) ratios for PPAS-6 and PPAS-3 scales were 6 and 3, respectively. A wide range of communality existed, with estimates ranging from .37 to .67. For excellent statistical power, we required a minimum sample of 50 respondents for a one-factor six- indicator solution and 110 respondents for a one-factor three-indicator model. Preliminary analyses included descriptive statistics of the six autonomy support items in the derivation subset. Inter-item correlations were derived as an initial assessment of *unidimensionality*. The latter is a subtype of construct validity indicating that only one construct is assessed by the items comprising a scale that is intended to measure a unitary trait. We also performed Horn’s parallel analysis and Velicer’s minimum average partial (MAP) test [83] on the six-item reference scale to further assess the unidimensionality aspect of its construct validity.

We utilized item response theory analysis,[84] specifically Samejima’s graded response model (GRM),[85] to calibrate the six polytomous items, derive item-level discrimination/difficulty indices plus Eigenvalues, and plot option characteristic curves (OCCs) as well as item/test information functions. The GRM yields a single slope (discrimination, α) parameter and (*n*– 1) threshold (or location or difficulty, *Ƅ*) parameters for polytomous items with *n* response categories. Items with higher (steeper) slope (α) are more efficient at discriminating among respondents with similar levels of the assessed trait/construct (θ). As a rule of thumb, α parameters from 0.65–1.34 indicate “moderate,” between 1.35–1.75 “high,” and >1.76 “very high” discrimination.[86] We ranked items by order of magnitude of α parameters. An item threshold (*Ƅ*) parameter is the point at which a respondent with a latent trait (θ) has equal (50:50) probability of endorsing a specific response option versus another (e.g. “none of the time” vs. “most of the time”). We utilized *Ƅ* values to indicate where on the autonomy support “trait” the respondents are most likely to endorse different item response options. A higher *Ƅ* value indicates a more difficult response option for respondents to endorse. Option characteristic curves and item information functions were used to assess the three best items to incorporate into a PPAS-3 scale. Item-level goodness-of-fit in the GRM was assessed using the generalized S-Σ^2^ index,[87] evaluated at the 1% significance level due to the short length of the scales.[88] To test whether *local independence* (i.e. the assumption that only the latent trait being measured, not any other variable, influences respondents’ ratings of each item) was tenable, we examined the (approximately) standardized local dependence (LD) Σ^2^ statistic [89] for each item pair. LD statistics < |5| are considered small or inconsequential, between |5| and |10| moderate or questionable, and > |10| as large, indicating leftover residual variance not accounted for by the IRT model. Item selection for inclusion in the short-form scale was further based on item loadings [90] on the full-length scale, item-to-scale correlations, and reliabilities of individual items.[91]

To compare construct validity of the PPAS-6 and PPAS-3 scales, we utilized exploratory structural equation modeling (ESEM) [92, 93] and confirmatory factor analysis (CFA).[94] Based on the classical rule of thumb of a 10:1 ratio of cases to free parameters in CFAs,[95] with 18 free parameters in the six-indicator model and nine free parameters in the three-indicator model, survey respondents exceeded by far the minimum sample required for sufficient statistical power. We evaluated the ESEM/CFA models’ goodness of fit using the comparative fit index (CFI), Tucker-Lewis Index (TLI), and the root mean square error of approximation (RMSEA) with its 90% confidence interval.[96] We assessed internal consistency for PPAS-6 and PPAS-3 scales using Cronbach’s coefficient alpha,[97] ordinal coefficient alpha,[98] composite (congeneric) reliability,[99] and average variance extracted.[81] We also performed regression analyses to further establish the two scales’ concurrent validity (i.e., how well one test can replace another test in instances whereby data on both index and criterion measures are collected at the same time). In a generalized univariate logistic regression with high autonomy support on the PPAS-6 scale as the outcome and the continuous PPAS-3 score as the predictor, we assessed the brief scale’s ability to discriminate physicians with high (≥ 3) ratings of autonomy support on the full-length scale. We examined the receiver operating characteristic (ROC) plot to explore cutoff points for ‘high’ autonomy support on the index scale that would maximize accuracy in discriminating high scores on the reference scale. Using the selected threshold to categorize ‘high’ autonomy support on the brief scale, we then tested inter-rater reliability between PPAS-6 and PPAS-3 scales based on percent agreement in rating ‘high’ autonomy support, Cohen’s kappa coefficient, Fleiss’ kappa statistic, Conger's kappa statistic, Gwet’s AC_1_ coefficient, Krippendorf’s alpha coefficient, and Brenann-Prediger coefficient.[100]

To obtain support for the scales’ external validity and generalizability we tested measurement invariance by comparing ESEM/CFA model fit within subgroups [101] of the validation population categorized by: (1) academic affiliation: defined as faculty versus non-faculty physicians; (2) clinical experience (a proxy for professional rank/seniority): coded as high- (≥20 years) versus low-experience (<20 years post-residency); (3) patient panel size: large (≥2,500) versus small panels (<2,500 active patients); (4) practice size: small practices (≤10 physicians) versus others; (5) medical specialty (internist, family practitioner or pediatrician); and (6) healthcare market (California, Massachusetts, or Upstate New York).

We derived means/medians of indices such as the coefficient of variation (CV_wg_),[102] average deviation (AD_M(J)_),[103] r*_wg(J),_[104] and a_wg(J),_[105] plus intra-class correlation coefficients ICC_(1)_ and ICC_(2)_,[106] to quantify the interrater agreement in PPAS-6/PPAS-3 scale scores at the subgroup level. Finally, we conducted within-and-between analysis (WABA) [107] to assess the variability (η^2^) [108] in scale scores nested within versus between subgroups.

We conducted most analyses using Mplus^®^ version 7.4 [109] and SAS^®^ version 9.4 (SAS Inc, Cary, NC). Item-fit and LD statistics for the GRM were derived with the MIRT package [110] in R^®^ version 3.6.0 (R Development Core Team, Vienna, Austria), and group-level interrater reliability indices with the IRA module [111] in Stata^®^ version 15 (StataCorp LLC, College Station, TX).

## Results

### Sample characteristics

General internists comprised the largest proportion among respondents from New York and Massachusetts; California respondents were predominantly family practitioners. Clinical experience and panel size demonstrated similar variability across sites: six of ten doctors had completed residency in the preceding 20 years or less; seven of ten had a panel of 2,500 or fewer patients. New York physicians differed in notable ways from the other respondents: (1) seven in ten had academic faculty appointments as compared to only five of ten in Massachusetts, two of ten in California; (2) eight in ten were in solo/small practices whereas more than nine of ten in both Massachusetts and California served in larger practices; and (3) on average had lower job control scores, higher ratings for task difficulty, and stronger beliefs that incentivized clinical guidelines hindered patient care. Three fourths of New York/California physicians rated themselves as satisfied with practice compared to six of ten in Massachusetts. See “Table 1” for further details regarding the characteristics of the study sample.

**Table 1:** Characteristics of the “Rewarding Results” study physicians and their practices.

		Total Sample	RIPA^a^	MHQP^b^	California
Number of respondents	N	**1,534**	**291**	**554**	**689**
Clinical specialty, n (%)	General Internist	627 (40.87)	120 (41.24)	290 (52.35)	217 (31.49)
	Family Practitioner	454 (29.60)	70 (24.05)	105 (18.95)	279 (40.49)
	General Pediatrician	369 (24.05)	97 (33.33)	127 (22.92)	145 (21.04)
	Other (e.g. Medicine-Pediatrics)	43 (2.80)	4 (1.37)	25 (4.51)	14 (2.03)
	Did not respond	41 (2.67)	0 (0.00)	7 (1.26)	34 (4.93)
Academic faculty status, n (%)	Has faculty appointment	602 (39.05)	197 (66.67)	269 (48.56)	136 (19.74)
	Has no faculty appointment	891 (58.08)	92 (31.62)	281 (50.72)	518 (75.18)
	Did not respond	44 (2.87)	5 (1.71)	4 (0.72)	35 (5.08)
Post-residency clinical experience, n (%)	> 20 Years	564 (36.77)	115 (39.52)	214 (38.63)	235 (34.11)
	≤ 20 Years	939 (61.21)	173 (59.45)	337 (60.83)	429 (62.26)
	Did not respond	31 (2.02)	3 (1.03)	3 (0.54)	25 (3.63)
Job satisfaction status, n (%)	Satisfied with Practice	1,082 (70.53)	222 (76.29)	344 (62.09)	516 (74.89)
	Not Satisfied with Practice	410 (26.73)	64 (21.99)	204 (36.82)	142 (20.61)
	Did not respond	42 (2.74)	5 (1.72)	6 (1.08)	31 (4.50)
Job satisfaction rating, median (Q_1_, Q_3_)	Score on 7-point rating scale	5 (4, 6)	5 (5, 6)	5 (4, 6)	5 (5, 6)
Physician attitude scores, mean (standard deviation)	Job Control	3.00 (.87)	2.72 (.84)	2.96 (.86)	3.16 (.85)
	Peer/Staff Cooperation	3.02 (.80)	2.94 (.67)	2.98 (.78)	3.08 (.86)
	Fairness of incentive distribution	2.99 (.97)	2.88 (.92)	2.91 (.93)	3.12 (1.02)
	Perceived hindrance to patient care	2.62 (.99)	2.81 (1.01)	2.57 (.96)	2.58 (1.01)
	Difficulty of clinical tasks	2.49 (.95)	2.74 (.92)	2.49 (.97)	2.38 (.93)
Practice/Office Size, n (%)	< 10 Practitioners	243 (15.97)	228 (78.35)	8 (1.44)	9 (1.31)
	≥ 10 Practitioners	1,284 (83.70)	61 (20.96)	543 (98.01)	680 (98.69)
	Did not respond	5 (0.33)	2 (0.69)	3 (0.54)	0 (0.00)
Patient Panel Size, n (%)	> 2,500 Active Patients	374 (24.38)	87 (29.90)	125 (22.56)	162 (23.51)
	≤ 2,500 Active Patients	1,098 (71.58)	196 (67.35)	413 (74.55)	489 (70.97)
	Did not respond	52 (4.04)	8 (2.75)	16 (2.89)	38 (5.51)

^a^Rochester Independent Practice Association

^b^Massachusetts Health Quality Partners

### Sample adequacy and number of extractable factors

Based on the derivation subsample, Bartlett’s test of sphericity was highly significant (Chi-square = 1848.5064; p < .0001). We thus reject the null hypothesis of no extractable factors and accept the alternative hypothesis that relations between study measures are strong enough to suggest the existence of underlying factors, i.e. their correlation matrix significantly differs from an identity matrix. The KMO test had a value of 0.864, with scores for the items ranging from 0.831 (item Q47) to 0.908 (item Q50), all exceeding the 0.800 threshold [80] indicative of adequacy for factor analysis.

The smallest average squared partial correlation yielded by Velicer’s MAP test in the derivation sample was 0.0503, and the Eigenvalue of the first component (3.499), which by itself accounted for almost 100% of the total variance, was almost five times that of a second component (0.707). This suggests that there is negligible residual variance after the first factor is extracted. Parallel analysis also indicated that only one factor with an Eigenvalue exceeding those generated by random data was extractable from the observed data. Thus, the MAP test and parallel analysis results both support the assumption of *unidimensionality* among PPAS items.

### Item scores distribution, inter-correlations, and reliability

Mean scores on the six PPAS items ranged from a low of 2.428 (SD .932) on Q49 to a high of 3.302 (SD .957) on Q47. Scores covered the entire range on the rating scale from 1 (‘none of the time’) to 5 (‘all of the time’). Respondents’ extent of agreement with each item varied somewhat by geography. Among New York physicians, items Q46, Q47 and Q50 skewed heavily toward agreement that ‘most’ or ‘all of the time’ the HMO sought good relations with physicians, wanted them to provide good patient care, and was confident in their ability to do so. Among California and Massachusetts participants, only item Q47 demonstrated a similar skew. In the latter two states, items Q48r and Q49 were heavily skewed toward agreement that ‘rarely’/‘none of the time’ does the HMO refrain from interfering in patient care or portray an understanding of physicians’ needs. “Table 2” details distributions of item response categories by study site.

**Table 2:** Frequencies of the six ‘Perceived autonomy support’ items by physician subpopulations in the full sample.

Item		Percentage of Item Response Category in Each Subpopulation														
No.	Response	*1 = None of the time*			*2 = Rarely*			*3 = Some of the time*			*4 = Most of the time*			*5 = All of the time*		
	Statement	RIPA^a^	MHQP^b^	CAL^c^	RIPA	MHQP	CAL	RIPA	MHQP	CAL	RIPA	MHQP	CAL	RIPA	MHQP	CAL
Q46	Health plan seeks to maintain good relationships with practitioners	4.48	7.66	6.60	7.59	22.63	22.43	17.93	34.12	35.19	59.31	33.39	31.67	10.69	2.19	4.11
Q47	Health plan wants me to take good care of my patients	2.77	6.17	5.72	5.54	20.51	17.45	19.72	27.95	30.79	62.98	42.11	41.94	9.00	3.27	4.11
Q48r	Health plan does not interfere with how I care for my patients	14.71	11.07	12.32	15.73	41.56	38.71	22.49	29.76	26.98	21.72	16.70	19.79	20.00	0.91	2.20
Q49	Health plan understands my situation/needs as a practitioner	12.46	17.27	17.01	22.15	48.73	40.76	39.79	27.64	28.59	23.53	5.82	12.61	2.08	0.55	1.03
Q50	Health plan has confidence in my ability to offer high quality care	2.79	5.10	4.84	8.71	20.04	13.64	34.49	42.62	37.98	44.95	29.69	37.54	9.06	2.55	6.01
Q51	Health plan encourages my questions and feedback	4.50	12.04	10.95	13.15	26.64	23.67	35.29	45.62	43.34	39.79	15.33	19.38	7.27	0.36	2.66

^a^ Rochester Independent Practice Association

^b^ Massachusetts Health Quality Partners

^c^ California physicians

Inter-item polychoric correlations ranged from a low of 0.342 (SE 0.037) between items Q48r and Q50 to a high of 0.701 (SE 0.023) between items Q46 and Q47. These were the three top-ranking items in point-biserial correlation with the PPAS-6 scale: Q49 (*r* = 0.745), Q46 (*r* = 0.727), and Q47 (*r* = 0.669). These three items, in the same rank order, would also lead to the largest decreases in internal consistency (as assessed by Cronbach’s α) were they to be deleted from the PPAS-6 scale. “Table 3” details inter-item and item-to-scale point-biserial correlations. Collectively these findings suggest that, of the six items available, Q49, Q46, and Q47 would be the best choices to incorporate into a PPAS-3 scale.

**Table 3:** Distributions and correlations of the six ‘Perceived Autonomy Support’ items in the derivation subsample.

Item #	Measures of Item Dispersion			Inter-Item Polychoric Correlation (Standard Error)						Biserial Correlation with 6-Item Scale	Biserial Correlation with 3-Item Scale	Cronbach’s Alpha^α^ of 6-Item scale with the Item Deleted	Cronbach’s Alpha^α^ of 3-Item scale with the Item Deleted
	Mean (SD)	Skewness	Kurtosis	Q46	Q47	Q48r	Q49	Q50	Q51				
Q46	3.125 (.994)	-0.452	-0.531	1.000						.727	.736	.815	.717
Q47	3.302 (.957)	-0.639	-0.285	**.701** (.023)	1.000					.669	.686	.826	.770
Q48r	2.602 (.964)	0.181	-0.770	**.468** (.033)	**.505** (.032)	1.000				.554		.847	
Q49	2.428 (.932)	0.286	-0.481	**.636** (.026)	**.603** (.028)	**.560** (.029)	1.000			.745	.648	.812	.805
Q50	3.243 (.913)	-0.405	-0.122	**.513** (.031)	**.554** (.030)	**.342** (.037)	**.580** (.029)	1.000		.586		.841	
Q51	2.834 (.938)	-0.136	-0.479	**.592** (.028)	**.539** (.031)	**.352** (.036)	**.612** (.027)	**.488** (.032)	1.000	.578		.843	

SD = standard deviation

α: Cronbach’s alpha is .855 for the full six-item scale, and .830 for the shortened three-item scale.

All correlations have p values < .0001

Floor effects are .8% and 2.8%, while ceiling effects are .1% and .9% for raw scores on the 6- and 3-item scales, respectively.

### Graded Response Model (GRM) analyses

**Fig 1:**
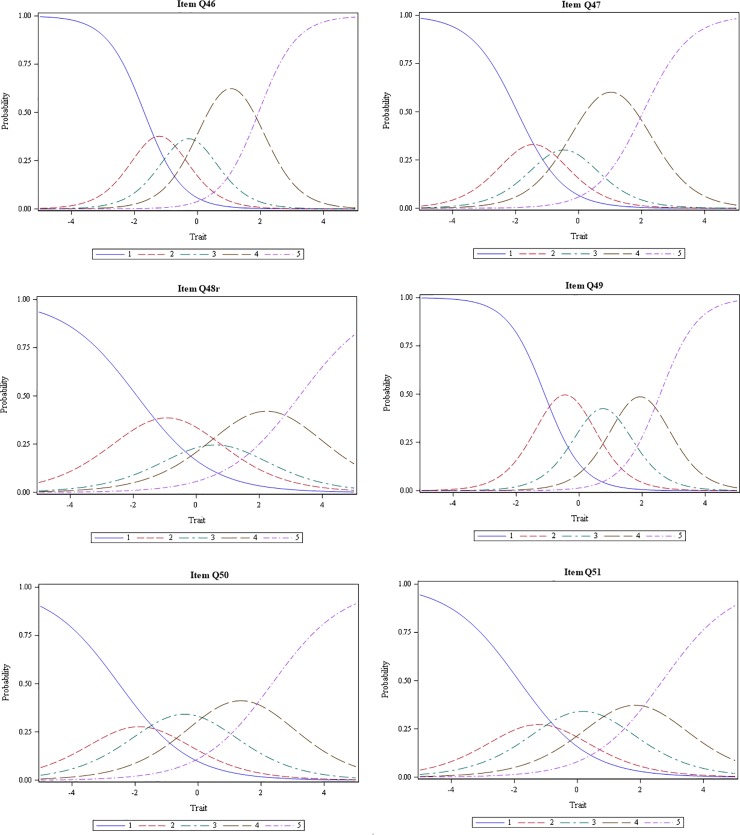
Option category characteristic curves for autonomy support items.

“Fig 1” reports the option characteristic curves (OCCs) for the six autonomy support items. Participants endorsed a reasonably wide spread of response options (from 1 to 5) on all six items. Probability of endorsing high-frequency extreme options 4 or 5 increased as the x-axis trait (autonomy support) rose, and that of low-frequency options 1 or 2 decreased as the level of autonomy support fell, which indicates *monotonicity* (i.e. the likelihood of endorsing a response option that represents more of the trait increases as a respondent’s actual level of the trait rises). This suggests that the items validly capture physicians’ perceptions of autonomy support from the payer organization. Items Q46 and Q47 had relatively higher endorsements of options of 4 and 5. Item Q49 showed the most equitable spread of endorsements across all five response options.

**Fig 2:**
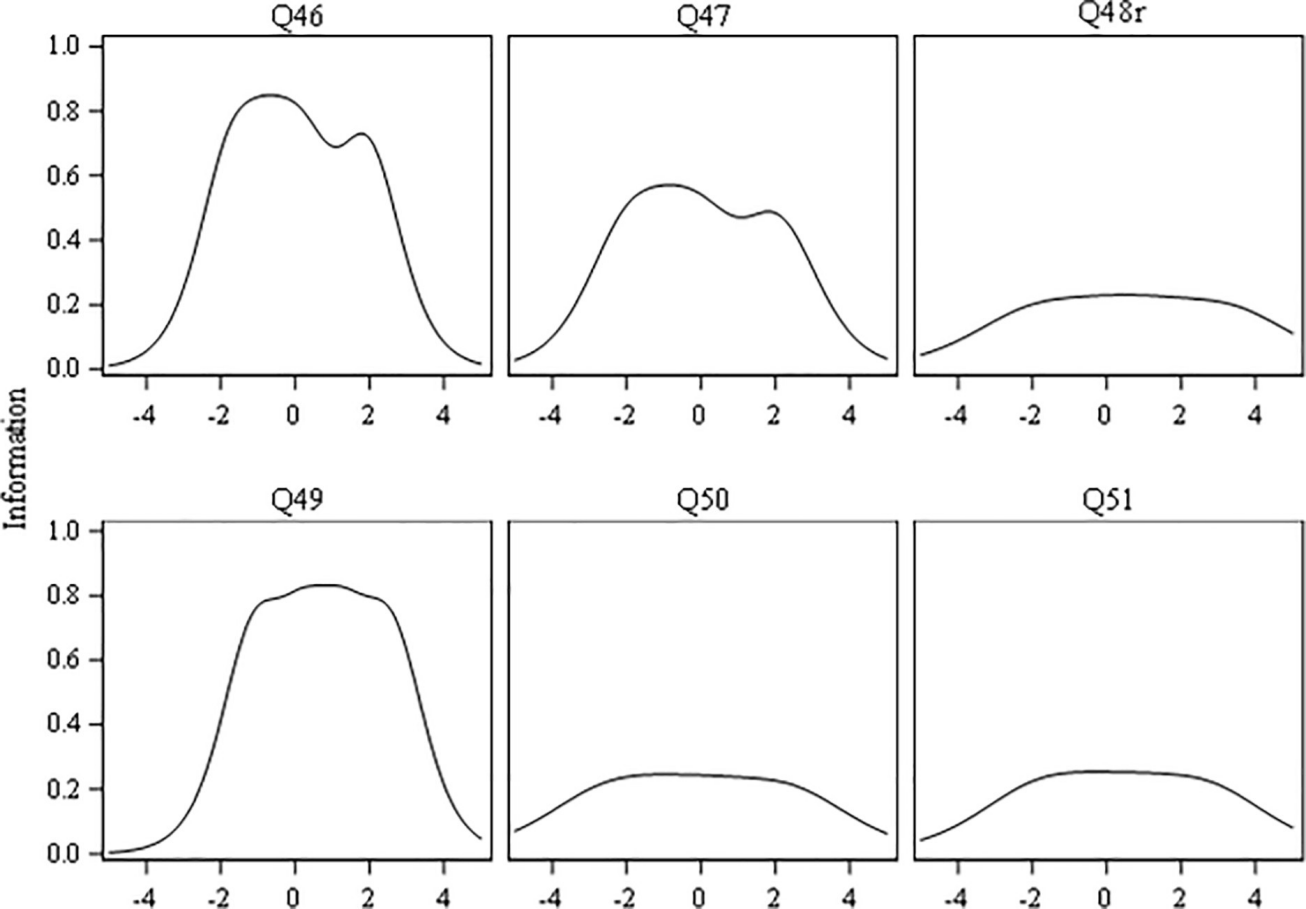
Information functions for the six autonomy support items.

**Fig 3:**
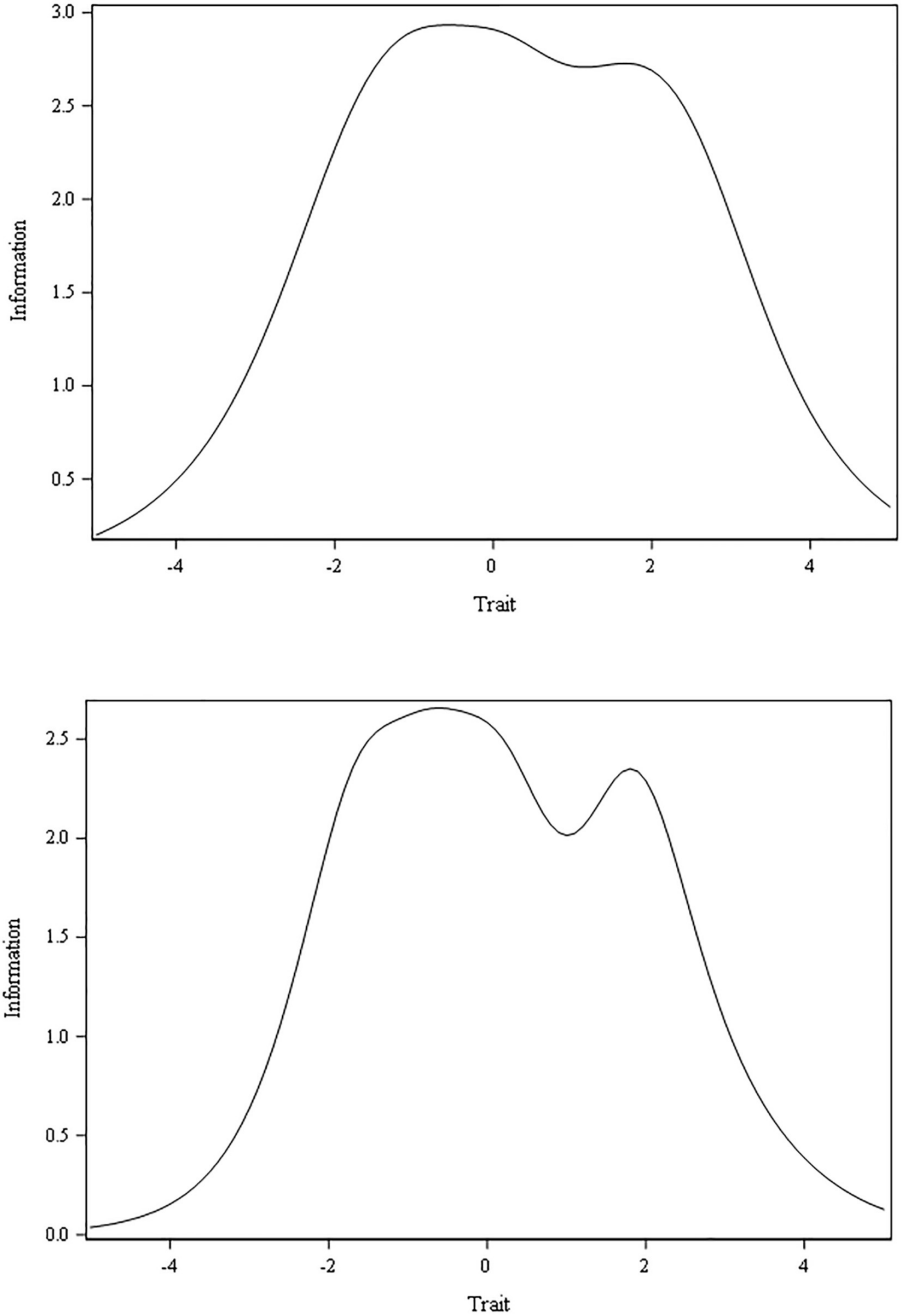
Test information functions for the six- and three-item scales. (A) PPAS-6 Scale. (B) PPAS-3 Scale.

“Table 4” outlines computations of the slopes (α, discrimination) and threshold (*Ƅ*_1_ to *Ƅ*_4_, difficulty or location) parameters for the six autonomy support items from the GRM analysis. Items Q46, Q47, and Q49 had “high” slopes (≥ 1.35); while items Q48r, Q50 and Q51 had “moderate” slopes (≥ 0.65 and ≤ 1.34). Largest, second, and third largest slopes were for Q49, Q46, and Q47, respectively. Thus, item Q49 was the most effective in discriminating between scores of individual physicians on the PPAS-6 scale. Q46 and Q47 were the second and third most effective, respectively. The GRM showed no evidence of local dependence: (1) no items had extremely high slopes (e.g. > 4) relative to others; and (2) standardized LD Σ^2^ statistics for all the item pairs were small (i.e. less than |5|). As for item fit, the S-Σ^2^ index (see “Table 4”) showed satisfactory fit for every item (all p values > .010), and ranged from 56.33 for item Q48r (highest) to 28.30 for Q49 (lowest). “Fig 2” shows plots of the item information functions. Of the six items, Q49, Q46, and Q47 contained the most “psychometric information” on autonomy support across the entire breadth of its variability. By contrast, items Q48r, Q50, and Q51 displayed flatter information functions. These findings further support the preferential selection of three items–Q49, Q46, and Q47 –for inclusion in a PPAS-3 scale. The significant (p < .001) change in the likelihood ratio Σ^2^ test [112] between 3- and 6-item unidimensional models indicates that the additional information in the PPAS-6 (versus PPAS-3) scale improves model-data fit. The test information functions (cf. “Fig 3”) show that the PPAS-6 provides greater psychometric information than the PPAS-3 scale.

**Table 4:** Item parameter estimates from the unidimensional graded response model on the derivation subsample.

Item #	Item Content	Slope	Response Category Threshold Estimates				Item Fit	
		α (s.e.)	*Ƅ*_*1*_ (s.e.)	*Ƅ*_*2*_ (s.e.)	*Ƅ*_*3*_ (s.e.)	*Ƅ*_*4*_ (s.e.)	S-Σ^2^	*p*
Q46	Health plan seeks to maintain good relations with practitioners	1.663 (.120)	-1.691 (.094)	-.737 (.060)	.180 (.052)	1.939 (.104)	37.350	.069
Q47	Health plan wants me to take good care of my patients	1.354 (.092)	-1.944 (.122)	-.928 (.070)	-.005 (.055)	2.052 (.116)	51.898	.011
Q48r	Health plan does not interfere with how I care for my patients	.858 (.060)	-1.884 (.133)	.014 (.069)	1.185 (.097)	3.276 (.238)	56.328	.013
Q49	Health plan understands my situation/needs as a practitioner	1.670 (.124)	-1.097 (.071)	.207 (.052)	1.296 (.075)	2.569 (.154)	28.297	.132
Q50	Health plan has confidence in my ability to offer high quality care	.887 (.061)	-2.156 (.167)	-1.232 (.098)	.374 (.071)	2.347 (.158)	54.483	.025
Q51	Health plan encourages my questions and feedback	.901 (.062)	-1.856 (.128)	-.614 (.076)	.964 (.086)	2.703 (.180)	41.898	.230

α is the item slope (discrimination) parameter; *b*_*1*_ to *b*_*4*_ are item response category threshold (difficulty, location) parameters; s.e. = standard error; S-Σ^2^ is the generalized item-level goodness-of-fit index: *p* is the significance level for the S-Σ^2^ index.

Model fit statistics: Akaike Information Criterion (AIC) = 10532.434; Bayesian Information Criterion (BIC) = 10671.434

Likelihood Ratio (LR) Σ^2^ / degrees of freedom (df) = 1901.462 / 15594; Log Likelihood = -5236.217

### Exploratory structural equation modeling on derivation subsample

Mardia’s tests for multivariate skewness and kurtosis were statistically significant, indicating that the multivariate normality assumption was violated. ESEM/CFA models were thus operationalized using the robust (mean- and variance-adjusted) weighted least squares (WLSMV), rather than maximum likelihood (ML), estimator.[113] ESEM of the one-factor six-item model indicated a good fit to the derivation subsample: CFI = .981; TLI = .968; RMSEA = .129 (90% CI = .109 –.150), p < .001. All six items had statistically significant factor loadings. The three items with the largest factor loadings, in order of ranking, were: Q46 (λ_standardized_ = .858, SE .012), Q49 (λ_standardized_ = .851, SE .013), and Q47 (λ_standardized_ = .807, SE .015). These three items also ranked highest in the proportion of their variation (R^2^) accounted for by the one-factor six-item model: .736 (SE .021), .724 (SE .023), and .651 (SE .024) respectively. ESEM findings thus supported preferential selection of Q46, Q49, and Q47, over the other three items, for incorporation into a shortened, PPAS-3 scale. The largest modification index (MI = 80.567) was for the correlation between items Q46 and Q47. No other MI exceeded 22.05. Item-to-item residual correlations in the single-factor six-item CFA model were below |.20|: the largest value (-0.079) was between items Q47 and Q51. The relatively low MIs and residual correlations provide further evidence that *local independence* was not violated.

### Exploratory structural equation modeling and confirmatory factor analyses on the validation subsample

ESEM/CFA models comparing goodness of fit between six- and three-item one-factor solutions in the validation subsample found adequate fit for both solutions. As expected for a single-factor model, ESEM and CFA (using the WLSMV estimator) yielded identical results. “Table 5” shows factor loadings in ESEM/CFA models for items of the PPAS-6 and PPAS-3 scales in the validation subsample. Item loadings onto a latent factor representing the PPAS-3 scale were significant and mirrored loadings onto one factor representing the full-length, six-item scale. Standardized coefficients for the six items of the PPAS-6 scale ranged from .825 for Q49 (highest) to .602 for Q48r (lowest). Standardized coefficients for the three items of the PPAS-3 scale ranged from .857 for Q46 to .742 for Q49. Respective proportions of variation (R^2^) in Q46, Q47, and Q49 accounted for by a single-factor solution were .660 (SE .024), .662 (SE .024), and .680 (SE .026) for the PPAS-6 scale; .738 (SE .029), .668 (SE .029), and .551 (SE .031) for the PPAS-3 scale. The PPAS-6 and PPAS-3 scales both manifested good overall fit (TLI > .95, CFI > .95) with the validation subsample, providing empirical evidence in favor of the two scales’ construct validity.

**Table 5:** Item Factor (λ) loadings and R^2^ values for single-factor ESEM and CFA models estimated on the validation subset.

Factor Model	Coefficients	Item Q46	Item Q47	Item Q48r	Item Q49	Item Q50	Item Q51
WLSMV CFA of 6-item PAS scale	Standardized	.812	.814	.602	.825	.659	.704
	Unstandardized (standard error)	.985 (.023)	.987 (.023)	.730 (.032)	1.000 (.000)	.799 (.028)	.854 (.024)
	R^2^ (standard error)	.660 (.024)	.662 (.024)	.362 (.029)	.680 (.026)	.434 (.029)	.495 (.027)
ESEM of 6-item PAS scale	Standardized	.812	.814	.602	.825	.659	.704
	Unstandardized (standard error)	.812 (.015)	.814 (.015)	.602 (.024)	.825 (.016)	.659 (.022)	.704 (.019)
	R^2^ (standard error)	.660 (.024)	.662 (.024)	.362 (.029)	.680 (.026)	.434 (.029)	.495 (.027)
WLSMV CFA of 3-item PAS scale	Standardized	.857	.818	-----	.742	-----	------
	Unstandardized (standard error)	1.154 (.041)	1.102 (.033)	-----	1.000 (.000)	-----	-----
	R^2^ (standard error)	.734 (.029)	.668 (.029)	-----	.551 (.031)	-----	-----
ESEM of 3-item PAS scale	Standardized	.857	.818	-----	.742	-----	-----
	Unstandardized (standard error)	.857 (.017)	.818 (.018)	-----	.742 (.021)	-----	-----
	R^2^ (standard error)	.734 (.029)	.668 (.029)	-----	.551 (.031)	-----	-----

### Convergent validity of the six-item and three-item scales

Respective polychoric correlations between scores on the PPAS-6 scale and measures of locus of job control, global satisfaction with practice, peer/staff cooperation, and fairness/equity of P4P-based practice guidelines, were: .323 (SE .032), .246 (SE .036), .199 (SE .036), .and .343 (SE .035). Polychoric correlations between the PPAS-3 scale and above-mentioned conceptually related constructs were: 313 (SE .033), .231 (SE .036), .169 (SE .036), and .327 (SE .036), respectively. The significant positive correlations support the convergent validity for both the PPAS-6 and PPAS-3 scales.

### Discriminant validity of the six-item and three-item scales

Polychoric correlations of the PPAS-6 scale with perceptions of P4P as hindering patient care and with clinical task difficulty were: -.274 (SE .036) and -.088 (SE .040), respectively. The PPAS-3 scale’s polychoric correlations with the two antonymous constructs were: -.236 (SE .037) and -.073 (SE .040), respectively. The significant negative correlations, especially with perceived hindrance to patient care, provide evidence in support of discriminant validity for the full-length and short-form PPAS scales.

### Internal consistency of the six-item and three-item scales

When their scores in the validation dataset are transformed to a percentile (0–100) format (respondent’s score / maximum score * 100) for easier comparability, respective means for the six- and three-item autonomy support scales are 48.03 (SD 17.85) and 48.74 (SD 20.35). Pearson’s correlation coefficient (r) between the PPAS-6 and PPAS-3 scales was .947 (p < .001), representing a shared variance of 89.68%. Cronbach’s coefficient α was .855 for the PPAS-6 scale and .830 for the PPAS-3 scale. Ordinal coefficient α was .874 and .847 for the PPAS-6 and PPAS-3 scales, respectively. Composite/congeneric reliabilities for PPAS-6 and PPAS-3 scales were .878 and .848, respectively; and AVEs were .549 and .651, respectively. These findings from disparate indices provide consistent empirical evidence of the internal consistency of both scales.

### Inter-rater reliability, sensitivity, and specificity of the six-item and three-item scales

We observed 89.79% agreement between PPAS-6 and PPAS-3 scales in discriminating high autonomy support. Inter-rater reliability in distinguishing high autonomy support was also indexed by: Cohen’s kappa (κ_c_) statistic .715 (SE .025); Fleiss’ Kappa Statistic (κ_f_) .713 (SE .026); Gwet’s AC_1_ coefficient .718 (SE .026); Brennan Prediger Coefficient (κ_3_) .716 (SE .025); and Krippendorff’s alpha (α) coefficient .713 (SE .026).

**Fig 4:**
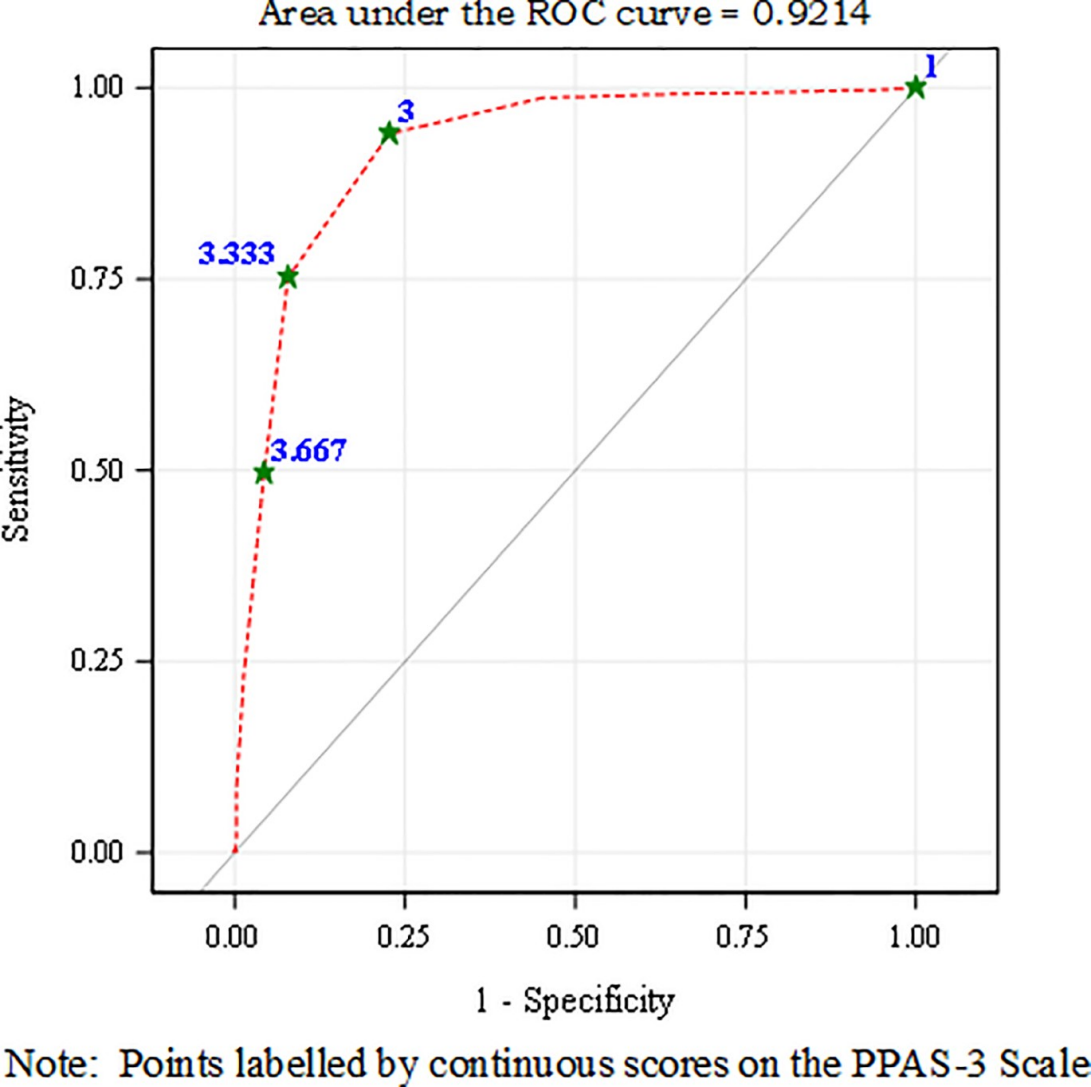
Receiver Operating Characteristic (ROC) plot showing ability of continuous scores on the three-item scale to distinguish high scores on the six-item scale.

Continuous scores on the PPAS-3 scale predicted high autonomy support on the PPAS-6 scale when assessed by a generalized univariate logistic regression (β = 3.682 [SE .268]; F_(df 751)_ = 189.46, p < .001; odds ratio 39.730 (95% CI 23.499–67.172). “Fig 4” shows the receiver operating characteristic (ROC) plot from this logistic regression. The area under the ROC curve is .921. The ROC plot suggests that an average score between 3.00–3.33 on the PPAS-3 scale is a good cut-off for delineating high autonomy support as assessed by equivalent scores ≥ 3 on the PPAS-6 scale. In another generalized univariate logistic regression, high scores (≥ 3) on the PPAS-3 scale strongly predicted high scores (≥ 3) on the PPAS-6 scale (β = 3.976 [SE .248]; F_(df 751)_ = 257.37, p < .001; odds ratio 53.292 (95% CI 32.731–86.769). Ability of high scores on the shorter scale to distinguish high autonomy support on the full-length scale is also evidenced by a sensitivity of 93.99%, specificity of 77.30%, positive predictive value of 81.08%, and negative predictive value of 92.56%.

### Measurement invariance across population subgroups

“Table 6” details goodness of fit indices for CFA models conducted among disparate subgroups of the validation population defined by geography, specialty, plus other demographic and practice characteristics. In subgroup models of the PPAS-6 scale, items Q49, Q46, and Q47 (not necessarily in that ranking order) consistently had higher loadings than the remaining items. Standardized coefficients for Q49, Q46 and Q47 ranged from .740 to .911; whereas similar loadings for other times were between .499 and .783. These findings corroborate derivation-sample CFA models and support the preferential selection of items Q49, Q46, and Q47 for incorporation into a PPAS-3 scale. The single-factor six- and three-indicator models all showed adequate fit to the data (CFI > .95, TLI > .95) for every population subgroup that we tested. For 12 of 14 physician subgroups tested, the CFI for the single-factor six-indicator CFA model, when compared to that derived on the whole validation subsample, did not change by a value exceeding ± .01.[101] That threshold was exceeded among two subgroups: PCPs in small practices, and/or serving in New York (ΔCFI = .02 for both). CFI for the one-factor three-indicator CFA model was identical among all subgroups. The item factor loadings and coefficients of variation for the CFA models of PPAS-6 and PPAS-3 among the subgroups are outlined in “S1 Table” in the supplement/appendix. The data indicated robust measurement invariance for both the PPAS-6 and PPAS-3 scales across the majority of subpopulations examined.

**Table 6:** Goodness of fit indices for confirmatory factor analyses estimated on subgroups of the validation subsample.

Population/Subpopulation Type		Measure Assessed by Uni-dimensional WLSMV CFA	Σ^2^	*df*	CFI	TLI	WRMR	RMSEA (90% CI)
Whole Sample	Validation Dataset	6-item scale	99.417	9	.981	.968	1.007	.115 (.095, .136)
		3-item scale	.000	0	1.000	1.000	.000	.000 (.000, .000)
Healthcare Market	California	6-item scale	57.309	9	.979	.965	.786	.123 (.094 - .155)
		3-item scale	.000	0	1.000	1.000	.000	.000 (.000, .000)
	MHQP	6-item scale	44.333	9	.968	.947	.761	.121 (.087 - .157)
		3-item scale	.000	0	1.000	1.000	.000	.000 (.000, .000)
	RIPA	6-item scale	*15*.*177*	9	0.995	0.992	0.368	.070 (.000 - .129)
		3-item scale	.000	0	1.000	1.000	.000	.000 (.000, .000)
Clinical Specialty	Internal Medicine	6-item scale	60.311	9	.971	.951	.818	0.135 (.104, .168)
		3-item scale	.000	0	1.000	1.000	.000	.000 (.000, .000)
	Family Practice	6-item scale	32.756	9	.979	.964	.619	.106 (.068, .146)
		3-item scale	.000	0	1.000	1.000	.000	.000 (.000, .000)
	Pediatrics	6-item scale	18.307	9	.993	.988	.449	.078 (.022, .129)
		3-item scale	.000	0	1.000	1.000	.000	.000 (.000, .000)
Faculty Status	Academic Faculty	6-item scale	43.130	9	.985	.974	.658	.115 (.082, .151)
		3-item scale	.000	0	1.000	1.000	.000	.000 (.000, .000)
	Non-faculty	6-item scale	72.056	9	.973	.955	.896	.124 (.098, .151)
		3-item scale	.000	0	1.000	1.000	.000	.000 (.000, .000)
Clinical Experience Post-residency	High (≥20 years)	6-item scale	49.893	9	.983	.972	.742	.124 (.091, .158)
		3-item scale	.000	0	1.000	1.000	.000	.000 (.000, .000)
	Low (<20 years)	6-item scale	53.022	9	.981	.968	.804	.104 (.078, .131)
		3-item scale	.000	0	1.000	1.000	.000	.000 (.000, .000)
Practice/Group Size	Small (5 or fewer practitioners)	6-item scale	13.405	9	.996	.993	.360	.065 (.000, .133)
		3-item scale	.000	0	1.000	1.000	.000	.000 (.000, .000)
	Medium/ Large	6-item scale	87.576	9	.978	.964	.955	.117 (.095, .140)
		3-item scale	.000	0	1.000	1.000	.000	.000 (.000, .000)
Patient Panel Size	Large (≥2,500 active patients)	6-item scale	24.762	9	.988	.980	.548	.096 (.052, .142)
		3-item scale	.000	0	1.000	1.000	.000	.000 (.000, .000)
	Small (<2,500 active patients)	6-item scale	76.153	9	.979	.965	.849	.117 (.094, .142)
		3-item scale	.000	0	1.000	1.000	.000	.000 (.000, .000)

Σ^2^ = chi-square test, CFI = comparative fit index; CI = confidence interval; CFA = confirmatory factor analysis; df = degrees of freedom; MHQP = Massachusetts Health Quality Partners; RIPA = Rochester Independent Practice Association (RIPA); RMSEA = root mean square error of approximation; TLI = Tucker-Lewis index; WLSMV = mean- and variance-adjusted weighted least squares estimator; WRMR = weighted root-mean-square residual

### Within-group variability in scores on the six-item and three-item scales

Within-subgroup coefficients of variation (CV_wg_) in PPAS-6 scale scores generally fell between 22 and 27%, and those for PPAS-3 scale scores were between 24 and 30%. The shorter scale, thus, showed only slightly more dispersion than the full scale within validation population subgroups. Within-subgroup interrater agreement was indexed by mean r*_WG(J)_ and a_WG_ values ranging from lows of .436 and .443 across specialties to highs of .565 and .551 across the three geographies, respectively. Mean AD_M(J)_ by subgroup ranged from a high of .859 across specialties to a low of .758 across geographies (states). The values satisfy significance thresholds enunciated by Dunlap *et al*.[114] By LeBreton and Senter’s [115] standards for the r_WG_ family of indices, derived r*_WG(J)_ values indicate *weak* to *moderate* levels of within-subgroup interrater agreement in scores on the six- and three-item scales. a_WG_ values, however, indicate unacceptably low interrater agreement across subgroups, based on Brown and Hauenstein’s [105] rules of thumb. To indicate strong agreement, AD_M(J)_ values for five-point scales should not exceed 0.83.[116] Mean AD_M(J)_ by specialty exceeded this value, and values for other subgroup categories were close.

ICC_(1)_ values for the PPAS-6 scale ranged from a low of .0010 across specialties and academic faculty categories to a high of .0123 across the three geographies; whereas values for the PPAS-3 scale ranged from a low of .0010 across panel size categories to a high of .0746 across practice/office size categories. This suggests that the proportion of variability in scale scores related to subgroup membership was minimal–ranging from 0.1% to 1.23% for PPAS-6, and 0.1% to 0.75% for PPAS-3. ICC_(2)_ values ranged from a low of .1242 across specialties to a high of .7541 across geographies for the PPAS-6 scale; from .2638 across panel size categories to .9673 across practice/office size categories for the PPAS-3 scale. Thus, subgroup mean scores on the scale scores varied widely in reliability (were reliable for some categories but not for others). WABA results consistently showed that the majority of the variability in scale scores was nested within subgroups versus between them. Within-subgroup eta-squared (η^2^_within_) ranged from .9890 (geographies) to .9981 (faculty status) for PPAS-6; and from .9518 (geographies) to .9981 (panel size categories) for PPAS-3 scores. Between-subgroup eta-squared (η^2^_between_) thus ranged from .0019 (faculty status) to .0110 (geographies) for PPAS-6; and from .0019 (panel size categories) to .0482 (geographies) for PPAS-3 scores. Whereas correlations of PPAS-6 and PPAS-3 scores with job control at the within-subgroup level (r_within_) were highly significant (p < .0001) according to both the R statistic and t-test, such correlations at the between-subgroup level (r_between_) were not significant (p>.05). “S2 Table” in the supplement/appendix outlines the key indices of within-subgroup variability and interrater agreement in PPAS-6 and PPAS-3 scores among the same validation population subgroups used to assess factor invariance. The collective evidence from these indices suggests that PPAS-6 and PPAS-3 scores are relatively independent and should not be aggregated into group-level measures but should be maintained as tools capturing individual-level ratings of autonomy support. “S1 Fig” in the supplement/appendix displays the distribution of scores on the PPAS-6 and PPAS-3 scales in the whole study sample.

## Discussion

Research increasingly documents the power of internal motivation to significantly influence work behavior. Autonomy support, as a key aspect of internal motivation, could potentially impel effective change in clinical behavior. Healthcare organizations can, for instance, have practitioners craft and implement their own QI projects rather than dictate pre-programmed approaches to them. Reliable and valid tools are thus critically needed to measure autonomy support, given its importance as an integral component of internal motivation, a potential gateway to improving clinical practice effectiveness/efficiency. Accordingly, we examined the psychometric validity/reliability of two versions of a new measure of physicians’ perceptions of autonomy support based on data from 1,534 respondents to our cross-sectional survey, who were serving in healthcare markets, situated in three different states, transitioning from volume- to value-based reimbursement of healthcare services. Our goals, in this study, were to assess reliability and validity of both regular-length (PPAS-6) and short-form (PPAS-3) versions of the scale, examine their potential generalizability across diverse physician subpopulations, and test appropriateness of utilizing the scales to capture group-level perceptions. Through a series of both classical test theory (CTT) and item response theory (IRT) analyses, we obtained sufficient evidence to confirm the factor structure of full-length and short-form versions of the scale. Both versions satisfied established criteria for construct validity, internal consistency, concurrent validity, inter-rater reliability, and measurement invariance.

The PPAS-3 includes items with wording that reflects sustenance of healthy relationships and mutual understanding between the payor organization and physicians as well as adequacy of support by the payor towards physicians’ volitional efforts to deliver good care. Additional items in the PPAS-6 include wording that reflects the extent to which the payor interferes with clinical decision-making, shows confidence in physicians’ clinical proficiency, and encourages their questions or feedback. The PPAS-3 scale thus represents interwoven relationships and/or healthy communication/connection between patients, providers, and payors, while the additional three items in the PPAS-6 scale appear to add content related to support for the autonomous conduct of healthcare procedures or processes. Analyses suggest that both measures capture autonomy support as a unidimensional rather than a multi-dimensional construct. Thus, PPAS-6 and PPAS-3 scales offer a broad, more global outlook on the overall autonomy support experienced by physicians. In addition to assessing autonomy support from the global or overarching perspective, these relatively short scales that we validated have the advantages of parsimony and low respondent burden. They are brief enough to be incorporated into questionnaires that combine multiple short scales assessing diverse constructs. They can also be administered repeatedly in longitudinal surveys and/or to large populations of physicians at relatively low cost. In contexts where concerns about limiting costs and/or respondent burden are outweighed by the need for deeper exploration of various facets of autonomy support, future studies should validate elongated versions of the PPAS scale by incorporating extra items to capture multiple dimensions of the construct. Items capturing support towards each of the three distinct facets of work autonomy (method autonomy, scheduling autonomy, and work criteria autonomy) proposed by Breaugh[117] and others,[118, 119] as well as towards social/economic work autonomy and administrative work autonomy,[59] which were not the focus of this study, could be tested as part of a multidimensional version of the scale.

Psychometrics of the measures we validated in the present study compare favorably with those of brief perceived autonomy support scales from various domains. For instance, a nine-item subscale of the Perceived Autonomy Support Scale for Employees (PASS-E), when rated by young Canadian healthcare professionals, had Cronbach’s α values of .90 for supervisors and .87 for colleagues.[56] In an adaptation of the problems at work (PAW) scale, comprising eight problem vignettes, to assess perceived autonomy support from managers, Baard *et al*. found that Cronbach’s α ranged from .66 for extremely controlling to .80 for highly autonomy-supportive managers.[73] The modified Health Care Climate Questionnaire (mHCCQ), a five-item version of the 15-item original scale, had a Cronbach’s α of .84 and correlation of .95 with the full scale in a smoking cessation study, and α of .80 and correlation of .91 with the full scale among diabetes patients, with factor analyses of its five items yielding a one-factor solution.[120] The measures emerging from the present study have the additional advantage of having been validated not just via CTT but using IRT analyses as well. Furthermore, given that systemic transitions from volume- to value-based reimbursement for clinician services are increasingly prevalent,[121] the PPAS-6 and PPAS-3 have the advantage of having been validated within such a context.

Our data indicated that the PPAS scales capture autonomy support ratings predominantly at the individual, and not at the collective/group, level. This suggests that the measures should not be utilized to assess how autonomy supportive the general clinical practice climate or setting is. They should instead be utilized to capture individual clinicians’ motivations and assessments of how much autonomy support each of them is experiencing from the payor, healthcare delivery organization, or some other entity that is expected to confer such support. Our data showed that the shorter PPAS-3 makes an accurate surrogate or proxy of the slightly longer PPAS-6. We propose that the PPAS-6 be considered the instrument of first resort because it yields significantly more psychometric information than the PPAS-3. However, in contexts where the concerns about limiting both costs/expenses and respondent burden are paramount, the PPAS-3 can be deployed with confidence because it is a good replacement for the longer PPAS-6.

### Limitations of the study and implications for future research

During data collection, physicians were located in organizations transforming their incentive architecture. It is unclear whether the factor structure of the measures may have been influenced by specific types of change processes that were underway or if the findings would have been different had no organizational transformation been occurring. While we adduced evidence favoring the potential generalizability of PPAS-6 and PPAS-3 to different physician subpopulations, extending the research to non-ambulatory care settings, such as long-term care or inpatient care, may be useful. Future work should also assess whether variable susceptibility to autonomy support perceptions (e.g. due to different sensory-processing sensitivities),[122] might be associated with differential item functioning in the PPAS measures. Our sample was limited to physicians and included no advanced-practice clinicians, such as nurse practitioners or physician assistants. Physicians increasingly practice in teams incorporating advanced practice clinicians,[123] and autonomy support is equally important to these allied professionals.[124] Future studies should validate our measures among nurse practitioners and physician assistants. The two versions of the PPAS scale could also be compared with alternative autonomy support measures in future studies to better examine convergent validity.

Due to the cross-sectional nature of our survey data, we were unable to assess the test-retest reliability of the measures. Future research should consider longitudinal or multi-panel tracking of autonomy support using PPAS measures to examine responsiveness of such ratings of autonomy support to diverse types of organizational transformations. Examining physicians’ ratings before and after a healthcare organization implements a change might reveal how well that change was implemented. Because payor organizations operate hierarchically above the physician, it could be argued that we captured *vertical* autonomy support.[46] Future work should assess the validity of the PPAS scale in capturing more *horizontal* autonomy support (e.g. from professional peers or even patients).[46, 56] Validity of the PPAS-6 and PPAS-3 measures when scored by trained interviewers instead of self-reported ratings via self-administered questionnaires, or when administered online or via social media platforms, should also be evaluated.

## Conclusions

Our findings demonstrated reliability, validity, and generalizability of the PPAS-6 and PPAS-3 measures among a tri-state population of physicians experiencing transformational changes associated with value-based pay-for-performance incentives. Further work to evaluate these measures in different practice contexts is necessary. The brief scales can help practice managers to validly and reliably assess and longitudinally track, at a relatively low cost and minimal respondent burden, the effect(s) of organizational changes on the autonomy support by specific, relevant entities towards individual physicians.

## Supporting information

S1 Table Item Factor (λ) Loadings and R^2^ values for CFA models estimated on subgroups of the validation subsample. (DOCX)

S2 Table Within-subgroup variability in the six- and three-item autonomy support scale scores. (DOCX)

S1 Fig Distribution of autonomy support scale scores in the full study sample. (A) Distribution of Scores on the Six-Item Scale. (B) Distribution of Scores on the Three-Item Scale.
(TIF)
