# Supplementary material for: Physicians’ perceptions of autonomy support during transition to value-based reimbursement: A multi-center psychometric evaluation of six-item and three-item measures
Source: PLoS One. 2020 Apr 1;15(4):e0230907. doi: 10.1371/journal.pone.0230907 (PMC7112234; doi:10.1371/journal.pone.0230907)
Supplement: S1 Table — (DOCX) [file pone.0230907.s001.docx]

**S1 Table: Item Factor (λ) Loadings and R^2^ Values for CFA Models Estimated on Subgroups of the Validation Subsample**

|  | **Factor Model** | **Coefficients** | **Item Q46** | **Item Q47** | **Item Q48r** | **Item Q49** | **Item Q50** | **Item Q51** |
| --- | --- | --- | --- | --- | --- | --- | --- | --- |
| California Physicians | WLSMV CFA of 6-item PAS scale | Standardized | .829 | .811 | .703 | .828 | .666 | .677 |
|  |  | Unstandardized (standard error) | 1.001 (.030) | .980 (.034) | .849 (.040) | 1.000 (.000) | .805 (.041) | .818 (.039) |
|  |  | R^2^  (standard error) | .687 (.034) | .658 (.037) | .494 (.043) | .686 (.035) | .444 (.043) | .459 (.043) |
|  | WLSMV CFA of 3-item PAS scale | Standardized | .893 | .785 | ----- | .752 | ----- | ------ |
|  |  | Unstandardized (standard error) | 1.188 (.054) | 1.044 (.051) |  | 1.000 (.000) |  |  |
|  |  | R^2^  (standard error) | .797 (.043) | .616 (.048) | ----- | .565 (.043) | ----- | ------ |
| MHQP Physicians | WLSMV CFA of 6-item PAS scale | Standardized | .742 | .776 | .521 | .741 | .610 | .637 |
|  |  | Unstandardized (standard error) | 1.001 (.062) | 1.046 (.057) | .703 (.073) | 1.000 (.000) | .822 (.061) | .860 (.053) |
|  |  | R^2^  (standard error) | .550 (.048) | .602 (.044) | .271 (.045) | .550 (.055) | .372 (.070) | .406 (.046) |
|  | WLSMV CFA of 3-item PAS scale | Standardized | .781 | .821 | ----- | .611 | ----- | ------ |
|  |  | Unstandardized (standard error) | 1.127 (.120) | 1.343 (.103) |  | 1.000 (.000) |  |  |
|  |  | R^2^  (standard error) | .609 (.065) | .674 (.058) | ----- | .374 (.056) | ----- | ------ |

|  | **Factor Model** | **Coefficients** | **Item Q46** | **Item Q47** | **Item Q48r** | **Item Q49** | **Item Q50** | **Item Q51** |
| --- | --- | --- | --- | --- | --- | --- | --- | --- |
| RIPA Physicians | WLSMV CFA of 6-item PAS scale | Standardized | .852 | .880 | .502 | .907 | .676 | .783 |
|  |  | Unstandardized (standard error) | .939 (.043) | .970 (.041) | .553 (.065) | 1.000 (.000) | .745 (.054) | .863 (.038) |
|  |  | R^2^  (standard error) | .660 (.024) | .662 (.024) | .362 (.029) | .680 (.026) | .434 (.029) | .495 (.027) |
|  | WLSMV CFA of 3-item PAS scale | Standardized | .857 | .818 | ----- | .742 | ----- | ------ |
|  |  | Unstandardized (standard error) | 1.154 (.041) | 1.102 (.033) |  | 1.000 (.000) |  |  |
|  |  | R^2^  (standard error) | .734 (.029) | .668 (.029) | ----- | .551 (.031) | ----- | ------ |
