# Supplementary material for: Physicians’ perceptions of autonomy support during transition to value-based reimbursement: A multi-center psychometric evaluation of six-item and three-item measures
Source: PLoS One. 2020 Apr 1;15(4):e0230907. doi: 10.1371/journal.pone.0230907 (PMC7112234; doi:10.1371/journal.pone.0230907)
Supplement: S2 Table — (DOCX) [file pone.0230907.s002.docx]

**S2 Table: Within-Subgroup Variability in the Six- and Three-item Autonomy Support Scale Scores**

| **Category** | **Subgroup** | **Six-Item (PPAS-6) Scale Scores** | | | | | | **Three-Item (PPAS-3) Scale Scores** | | | | | |
| --- | --- | --- | --- | --- | --- | --- | --- | --- | --- | --- | --- | --- | --- |
|  |  | r*_WG(J)_ Index | **a**_WG_ Index | average deviation (AD_M(J)_) | ICC_(2)_ | η^2^_betw_ | η^2^_with_ | r*_WG(J)_ Index | **a**_WG_ Index | average deviation (AD_M(J)_) | ICC_(2)_ | η^2^_betw_ | η^2^_with_ |
| Study Site | California | .5652 | .5507 | .7575 | .7541^a^ | .0110 | .9890^c^ | .5566 | .5378 | .7583 | .9465^b^ | .0482 | .9518^c^ |
|  | MHQP |  |  |  |  |  |  |  |  |  |  |  |  |
|  | RIPA |  |  |  |  |  |  |  |  |  |  |  |  |
| Clinical Specialty | Internist | .4360 | .4425 | .8585 | .1242^a^ | .0062 | .9938^c^ | . 3942 | .3953 | .9049 | .3079^a^ | .0078 | .9922^c^ |
|  | Family Practitioner |  |  |  |  |  |  |  |  |  |  |  |  |
|  | Pediatrician |  |  |  |  |  |  |  |  |  |  |  |  |
| Faculty Status | Faculty | . 5482 | .5357 | .7828 | .2741^b^ | .0019 | .9981^c^ | .5352 | .5184 | .8028 | .3258^b^ | .0020 | .9980^c^ |
|  | Nonfaculty |  |  |  |  |  |  |  |  |  |  |  |  |
| Practice Size | ≤10 physicians | . 5591 | . 5435 | .7622 | .6896^b^ | .0044 | .9956^c^ | .5606 | .5394 | .7506 | .9673^b^ | .0401 | .9599^c^ |
|  | >10 physicians |  |  |  |  |  |  |  |  |  |  |  |  |
| Patient Panel Size | ≥ 2,500 | . 5336 | .5201 | .7954 | .5456^b^ | .0031 | .9969^c^ | .5241 | .5062 | .8124 | .2638^a^ | .0019 | .9981^c^ |
|  | < 2,500 |  |  |  |  |  |  |  |  |  |  |  |  |
| Years Post-residency | ≥ 20 years | . 5372 | .5250 | .7932 | .6419^a^ | .0038 | .9962^c^ | .5262 | .5096 | .8103 | .8181^a^ | .0074 | .9926^c^ |
|  | < 20 years |  |  |  |  |  |  |  |  |  |  |  |  |

ICC = Intraclass Correlation Coefficient;

a: Levene’s F test is significant (p <.05), i.e. we can reject the null hypothesis (H_0_) of within-subgroup homogeneity of variance

b: Levene’s F test is non-significant (p >.05), i.e. we fail to reject the null hypothesis (H_0_) of within-subgroup homogeneity of variance

c: Correlation between this scale and job control at the within-subgroup level (r_within_) is significant (p<.05) according to both the R test and t-test, whereas such correlation at the between-subgroup level (r_between_) is not significant.
