# Supplementary figures and images for: Physicians’ perceptions of autonomy support during transition to value-based reimbursement: A multi-center psychometric evaluation of six-item and three-item measures
Source: PLoS One. 2020 Apr 1;15(4):e0230907. doi: 10.1371/journal.pone.0230907 (PMC7112234; doi:10.1371/journal.pone.0230907)

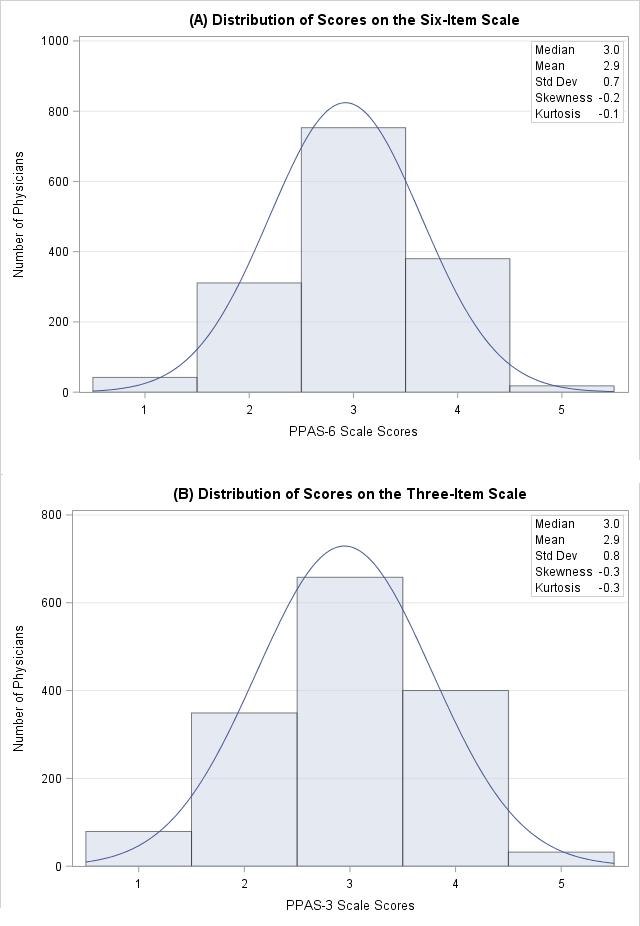

Supplement: S1 Fig — (A) Distribution of Scores on the Six-Item Scale. (B) Distribution of Scores on the Three-Item Scale. (TIF) [file pone.0230907.s003.tif]
